# Comparing Complications and Patient Satisfaction Following Injectable Collagenase Versus Limited Fasciectomy for Dupuytren’s Disease: A Systematic Review and Meta-Analysis

**DOI:** 10.7759/cureus.53147

**Published:** 2024-01-29

**Authors:** Zainah A Alhebshi, Aya O Bamuqabel, Zainab Alqurain, Dana Dahlan, Hanan I Wasaya, Ziyad S Al Saedi, Gutaybah S Alqarni, Danah Alqarni, Bayan Ghalimah

**Affiliations:** 1 College of Medicine and Surgery, Batterjee Medical College, Jeddah, SAU; 2 College of Medicine, Faculty of Medicine, Umm Al-Qura University, Makkah, SAU; 3 College of Medicine, Faculty of Medicine, University of Jeddah, Jeddah, SAU; 4 College of Medicine and Surgery, King Saud Bin Abdulaziz University for Health Sciences, Jeddah, SAU; 5 Department of Orthopaedic Surgery, Faculty of Medicine, King Abdulaziz University, Jeddah, SAU

**Keywords:** collagenase clostridium histolyticum injections, patient satisfaction, clinical outcomes, complications, injectable collagenase, partial fasciectomy, limited fasciectomy, joint contracture, dupuytren contracture, dupuytren's disease

## Abstract

Dupuytren's disease (DD) is a fibroproliferative disorder that manifests as an abnormal growth of myofibroblasts, causing nodule formation and contractures and affecting digit function. If left untreated, these contractures can lead to a loss of mobility and potentially impact hand function. This systematic review critically compares and evaluates the existing literature on the complications and patient satisfaction following injectable collagenase *Clostridium histolyticum* (CCH) versus limited fasciectomy (LF) for DD.

We performed a comprehensive search of the PubMed, Medical Literature Analysis and Retrieval System Online (MEDLINE), The Cochrane Library, and Excerpta Medica database (EMBASE) databases from 2006 to August 2023. This research targeted all clinical studies involving adults who underwent injectable collagenase and/or limited fasciectomy in the management of DD. Out of the 437 identified studies, only 53 were considered eligible for our analysis, and merely 14 met our inclusion criteria. These selected studies encompassed a total of 967 patients with 1,344 treated joints, with an average follow-up duration of 19.22 (ranging from one to 84.06) months. Within this cohort, 498 joints from 385 patients underwent LF, while 846 joints from 491 patients received CCH injections. Notably, among the 491 patients treated with CCH, 1,060 complications were reported, averaging 2.15 complications per patient, with the most common being contusion/bruising/hematoma/ecchymosis (22.54%), and edema/swelling (18.96%). In contrast, among the 385 patients treated with LF, only 97 complications were reported, translating to 0.25 complications per patient, with the most frequent being paraesthesia or numbness (23.7%), scar sequelae like skin laceration, tear, fissure, or hypertrophic scar (23.7%), and neuropraxia or nerve injury (22.6%). Our meta-analysis indicates that paraesthesia or numbness is more frequently observed in LF than CCH injections, although without statistical significance, with a risk ratio (RR) of 0.39 (95% confidence interval (CI) 0.13-1.18, p-value 0.1). However, scar sequelae (hypertrophic scar, skin laceration, tear, or fissure) show a contrasting pattern, being more commonly associated with CCH injections than LF, with an RR of 1.98 (95% CI 0.26-14.85, p-value 0.51), which, upon eliminating the source of heterogeneity, becomes statistically significant, with an RR of 4.98 (95% CI 1.40-17.72, p-value 0.01). Our data revealed a higher frequency of complications with CCH compared to LF, although more severe adverse effects were observed in the LF group, such as neuropraxia or nerve injury. Scar sequelae were more common with CCH injections. Despite both treatments showing increased patient satisfaction at the final follow-up, CCH injection resulted in earlier improvements in satisfaction.

## Introduction and background

Dupuytren's disease (DD), or palmar fibromatosis, is a frequently encountered hand surgery condition with a notable prevalence and significant impact on digit function [1]. The disease involves abnormal growth of myofibroblasts in the hand, with the fourth (ring) and fifth (small or pinky) digits being most frequently affected, particularly at the metacarpophalangeal (MCP) and proximal interphalangeal (PIP) joints [2, 3]. The myofibroblasts are predominantly composed of type III collagen and are influenced by cytokines such as interleukin-1 (IL-1) and transforming growth factor beta-1 (TGF-β1) [4]. The progression of Dupuytren contracture occurs in three phases: proliferative, involutional, and residual, with the proliferative stage characterized by fascial fibroplasia, nodule formation, and fibroblast proliferation [5]. Uncontrolled fibroblast proliferation, induced by local mediators, is implicated in nodule formation. The involution phase involves cell rearrangement along tissue stress lines, leading to cord development [6]. Myofibroblasts dominate this phase, contributing to collagen synthesis and contraction, resulting in nodule formation and subsequent contractures [1]. In the residual phase, nodules regress, leaving thick cords and relatively acellular residual tissue, causing visible cords and severe contractures [7, 8]. Dupuytren's disease shares a benign nature and similar etiology with related fibrous tissue disorders, such as Peyronie's disease, Ledderhose disease, and Garrod disease, all characterized by the development of fibrous tissue in various body parts [3].

The reported prevalence of DD varies from 2% to 42%, influenced mainly by ethnicity, gender, and age [1]. Numerous studies [1,5,9] have consistently reported an age-related increase in the prevalence of DD, with a higher occurrence in males and individuals of Northern European descent. Surprisingly, a 2020 study identified the highest DD rate in Africa at 17.2%. Furthermore, subgroup analysis demonstrated the greatest prevalence among patients with type 1 diabetes, reaching 34.1% [4]. In addition, a prospective study involving 600 adult Saudi patients revealed a remarkably low detection of DD in only two individuals (0.003%), both of Saudi descent, highlighting the rarity of DD in Saudi Arabians [9].

Treatment decisions revolve around the condition’s impact on a patient's quality of life, with the main goals of having a straight finger with a good arch of motion, minimizing recurrence, and preventing complications [10]. Non-operative treatment options include observation and occupational therapy [11]. On the other hand, operative management includes percutaneous needle fasciotomy (PNF), collagenase *Clostridium histolyticum* (CCH) injections, limited fasciectomy (LF), and dermofasciectomy (DF) [12]. Certainly, LF stands out as a surgical procedure due to its low complication rates and acceptable results [1]. It entails strategic incisions (transverse, Z-plasty, Bruner, or mid-lateral), followed by precise cord dissection to correct joint contractures. Sequential steps address persistent contracture, involving fascial release and ligament adjustments. This is followed by a post-correction assessment that includes perfusion and hemostasis checks, recording the correction degree, and evaluating joint functions. To end with closure that aims to avoid tension, postoperative care involves pain relief, elevation, hand therapy, and custom splints for scar remodeling [13, 14]. In contrast, CCH, sanctioned by the FDA in February 2010 and consisting of collagenase AUX-I and collagenase AUX II, represents an alternative approach [15]. These enzymes, derived from *Clostridium histolyticum*, have collagen-hydrolyzing properties and are employed to dissolve the collagen cords associated with DD. Collagenase *Clostridium histolyticum* provides a non-surgical solution administered in an office setting, demonstrating promising outcomes with minimal morbidity [16]. 

As outlined by Chen et al., while LF stands as the prevailing standard and the most widely employed treatment for DD, it is associated with a recurrence rate ranging from 12% to 39% over a mean follow-up duration of 1.5 to 7.3 years [17]. In comparison, Chen et al. contrasted this with CCH injections, revealing a lower recurrence rate of 10% to 31% and a mean follow-up time spanning 120 days to four years [17]. Hence, our systematic review and meta-analysis aimed to compare these treatments regarding complications and patient satisfaction, providing updated guidelines for clinicians to choose a safer and more effective approach to managing DD.

## Review

Methods and materials

Registration

This review was conducted in accordance with the protocol for the International Prospective Register of Systematic Reviews (PROSPERO) (ID: CRD42023457954) [18]. A systematic search was conducted in August 2023 using the following electronic databases: PubMed, Medical Literature Analysis and Retrieval System Online (MEDLINE), The Cochrane Library, and the Excerpta Medica database (EMBASE). An example of a search strategy for EMBASE was as follows: (Dupuytren OR Dupuytren’s disease OR contracture OR Dupuytren contracture OR joint contracture) AND (injectable collagenase OR collagenase OR CCH OR collagenase *Clostridium histolyticum*) AND (limited fasciectomy OR partial fasciectomy OR open partial fasciectomy) AND (complications OR clinical outcomes OR patient satisfaction OR satisfaction).

Study Selection

Four investigators (Alqarni, AlQurain, Alhebshi, and Wasaya) screened the titles and abstracts of the included articles and selected relevant studies for a full review. In case of any discrepancies, the article proceeded to a full-text review. Articles were extracted from the databases and screened using Rayyan Software (Rayyan Systems Inc., Cambridge, MA) [19]. The inclusion criteria for the systematic review were: (1) Studies published from 2006 to August 2023; (2) Studies that reported the number of people assessed; (3) Only studies published in English; (4) Study population including only adults; (5) Studies that used injectable collagenase and/or LF in the management of Dupuytren contracture; (6) Papers reporting DD in PIP and/or MCP joints; (7) The included studies reported outcomes of interest relevant to the clinical question; (8) Original articles (randomized controlled trials (RCTs), cross-sectional, prospective cohort, retrospective studies), case-control studies, case reports, and case series. The following criteria were excluded from this study: (1) Studies published in languages other than English; (2) Studies that did not use injectable collagenase and/or LF in the management of DD; (3) Papers not reporting DD in PIP or MCP joints; (4) Studies that did not report outcomes of interest for the clinical question; (5) Studies that used other management approaches; (6) Studies other than original articles (RCTs, cross-sectional, prospective cohort, retrospective studies), case-control studies, case reports, and case series.

Screening and Data Extraction

Full-text articles were retrieved and screened for eligibility. Discrepancies were discussed mutually, and the senior author (Alhebshi) was consulted for the final determination of the inclusion or exclusion of the article. Data from each report were retrieved by two investigators. Two authors (Alqarni and Al Saedi) screened the articles for our inclusion criteria and extracted the following information: (1) Study details: last name of the first author, year of publication, name of journal, country, study design; (2) Patients’ demographics, including age group and gender; (3) Intervention details: type of management (CCH injection and/or LF), follow-up period, total number of digits involved, number of the affected joints complications (infection, neurapraxia, paresthesia/numbness, scar sequelae as skin laceration, tear, fissure, or hypertrophic scar, etc.), and the number of patients who experienced them, patients satisfaction by using: Quick Disabilities of the Arm, Shoulder, and Hand (Quick DASH) score, Michigan hand Outcomes Questionnaire (MHQ) satisfaction score, Unité Rhumatologique des Affections de la Main (URAM) scale, EuroQol-visual analogue scales (EQ-VAS), and the Mean Hand 10 score; (4) Outcome measures: To compare and evaluate the complications and patient satisfaction following injectable collagenase versus LF for DD.

Risk of Bias and Quality Assessment

Two authors (Bamuqabel and Dahlan) evaluated the quality of the non-randomized studies that were included and assessed the risk of bias by using the methodological index for non-randomized studies (MINORS) tool [20]. This tool has two versions of assessment for comparative and non-comparative studies; eight and 12 fixed domains, respectively, were checked by the authors. Each domain was scored on a 0-2 scale, with a maximum score of 16 for non-comparative studies and 24 for comparative studies. On the other hand, the Revised Cochrane risk of bias (RoB 2) tool [21] was used for RCTs with five fixed domains. These domains concentrate on elements concerning the design, implementation, and depiction of the trial. A set of targeted inquiries referred to as signaling questions,' is employed to draw out details pertinent to bias risk. Subsequently, an algorithm is employed to assess these responses, resulting in evaluations categorized as 'low' (for all domains, the risk of bias is low), 'some concerns' (for at least one of the domains, there is some concern), or 'high' (for at least one domain, there is a high risk or some concerns for multiple domains).

Statistical Analysis

Statistical analysis was conducted using Review Manager (RevMan) Version 5.4, a software developed by the Nordic Cochrane Center and the Cochrane Collaboration in 2020. RevMan was employed for the calculation of risk ratios (RR) with 95% confidence intervals (CI). For the analysis of complications, specifically skin laceration/tear/fissure and paresthesia/numbness, event counts were recorded along with the total number of patients in each group. In cases where substantial heterogeneity was identified (I2 >50%), a random-effects model was adopted; conversely, a fixed-effects model was applied if heterogeneity was deemed minimal. A significance level of P <0.05 was considered the threshold for statistical significance.

Patient satisfaction

The importance of the patient's perspective in assessing DD treatments in the context of evidence-based medicine is recognized. Clinics now utilize patient-reported outcome measures (PROMs) such as Quick DASH, MHQ, URAM, EQ-VAS, and Mean Hand 10 to comprehensively evaluate treatment effectiveness, fostering patient-centered care. Quick DASH, an 11-item questionnaire, assesses arm, shoulder, and hand disabilities, with higher scores indicating more severe disability (up to 100) and a focus on streamlining assessment [22]. The MHQ evaluates various aspects of hand and upper extremity conditions, with our study emphasizing the satisfaction subdomain, where higher scores (up to 100) denote greater satisfaction and lower scores (down to zero) indicate lower satisfaction [23]. The URAM, a nine-item score, offers a concise tool for assessing physical disability, with scores ranging from zero (best) to 45 (worst) [24]. The EQ-VAS involves a vertical visual scale for patients to provide a global health assessment, ranging from zero (worst health) to 100 (best health) [25]. Hand 10 evaluates upper extremity function satisfaction using ten self-administered questions on a 0-10 scale, with a higher total score signifying worse function (0-100 range) and visual aids for clarity [26].

Results

An Overview of the Reviewed Studies’ Characteristics

**Figure 1:**
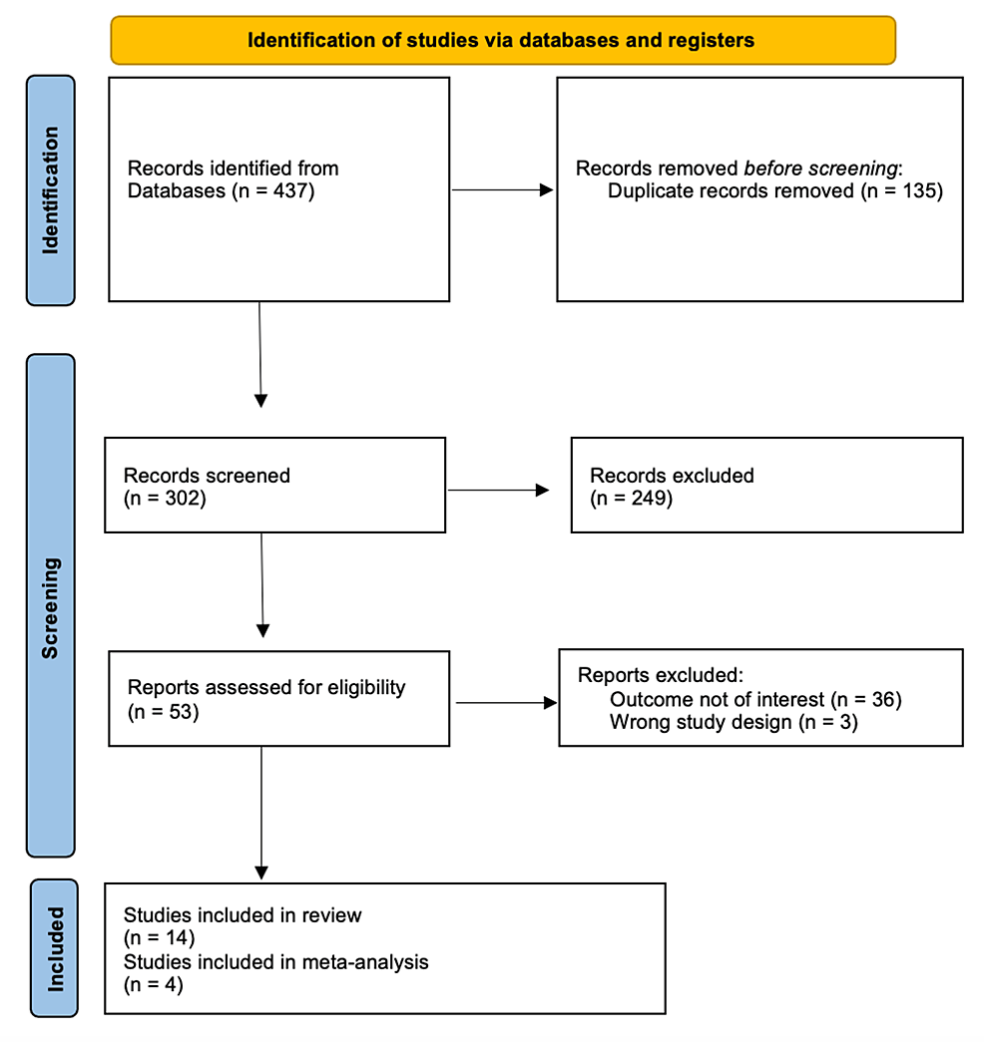
A flowchart outlining the selection of the reviewed studies according to the Preferred Reporting Items for Systematic Reviews and Meta-Analysis (PRISMA) n: number

A total of 53 studies underwent eligibility assessment from an initial pool of 437 identified records from the four main databases: PubMed, MEDLINE, The Cochrane Library, and Embase. Among them, 14 publications met the inclusion criteria, as depicted in Figure 1, representing a total of 967 patients with an average follow-up of 19.22 (1-84.06) months.

Of these 14 studies [26-39], six pertained to CCH injection [26, 35-39], two to LF [33, 34], and six directly compared these two treatment modalities [27-32], as summarized in Table 1.

**Table 1:** Characteristics of the included studies CCH: collagenase *Clostridium histolyticum*; LF: limited fasciectomy; RCT: randomized controlled trial; NR: not reported; No., n.: number; *one unreported gender

Authors/year and journal	Country	Study subjects	Females, n. (%)	Males, n. (%)	Mean age ± SD	Total no. of digits	Total no. of joints	Study type	Final follow-up duration	Type of operation
Kuboi et al., 2021 [26]	Japan	14	2 (14)	12 (86)	69.5	14	14	Prospective cohort	6 months	CCH
Yamamoto et al., 2022 [27]	Japan	78	3 (3.85)	75 (96.15)	70.66 ± 8.45	NR	307 (LF= 109, CCH= 198)	Prospective cohort	26 weeks	LF and CCH
Leclère et al., 2018 [28]	Switzerland	52	8 (15.38)	44 (84.62)	64 ± 10.4	68	118 (LF= 32, CCH= 86)	Retrospective cohort	24 months	LF and CCH
Tay et al., 2015 [29]	Malaysia	37	9 (24.3)	28 (75.7)	65.66 ± 8.75	NR	106 (LF= 77, CCH= 29)	Retrospective cohort	24 months	LF and CCH
Zhou et al., 2015 [30]	Netherlands	132	28 (21.2)	104 (78.8)	62 ± 9.076	201	177 (LF= 91, CCH= 86)	Prospective cohort	3 months	LF and CCH
Povlsen et al., 2014 [31]	United Kingdom	20	2 (10)	18 (90)	64.75	20	20 (LF= 10, CCH= 10)	Prospective cohort	3 months	LF and CCH
Naam et al., 2013 [32]	United States	46	10 (21.7)	36 (78.3)	65.91	NR	84 (LF= 52, CCH= 32)	Retrospective cohort	38 months	LF and CCH
Zhou et al., 2016 [33]	Austria	194	60 (30.9)	134 (69.1)	63 ± 9	NR	NR	Prospective cohort	12 months	LF
van Rijssen et al., 2006 [34]	Netherland	56*	10 (17.9)	45 (80.4)	64	NR	127	RCT	6 weeks	LF
Alberton et al., 2013 [35]	Italy	40	4 (10)	36 (90)	66	40	40	Prospective cohort	6 months	CCH
Abe et al., 2019 [36]	Japan	36	3 (8.3)	33 (91.7)	NR	NR	46	RCT	36 months	CCH
Sanjuan-Cerveró et al., 2018 [37]	Spain	151	19 (12.6)	132 (87.4)	66.05 ± 8.63	NR	194	Prospective cohort	1 month	CCH
De Vitis et al., 2020 [38]	Italy	45	7 (15.6)	38 (84.4)	66.4 ± 7.3	NR	45	Prospective cohort	7 years	CCH
Basso et al., 2023 [39]	Italy	66	9 (13.7)	57 (86.3)	64	66	66	Prospective cohort	24 months	CCH

Complications of CCH Versus LF in Comparative Studies

Among the six CCH injection studies, five were prospective cohort studies [26,35,37-39], while one was an RCT [36]. The two LF studies comprised both an RCT [34] and prospective cohort studies [33]. Half of the comparative studies were retrospective reviews [28-29,32], with the remaining being prospective in nature [27,30,31]. Essential details, such as the number of patients, mean patient ages, gender, and follow-up durations, were consistently reported across all studies.

For the comparative studies [27-32], the total number of joints considered was 812, involving 365 patients. Of these, five studies [27-31] provided information on complications, totaling 728 treated joints in 319 patients, with 135 patients undergoing LF and 184 receiving CCH injections. The total reported complications in these comparative studies numbered 220, with 32 complications associated with LF (14.54%) and 188 complications related to CCH injection (85.45%). The most frequently reported complications across these papers included edema/swelling (55 out of 220, 25%), contusion/bruising/hematoma (49 out of 220, 22.27%), scar sequelae as skin laceration, tear, fissure, or hypertrophic scar (29 out of 220, 13.18%), pain in extremities (17 out of 220, 7.72%), and paraesthesia/numbness (14 out of 220, 6.36%), as highlighted in Table 2.

**Table 2:** Complications of limited fasciectomy versus collagenase injections in the comparative studies CCH: collagenase *Clostridium histolyticum*; LF: limited fasciectomy; NR: not reported; (n): number

Articles	Yamamoto et al., 2022 [27]		Leclère et al., 2018 [28]		Tay et al., 2015 [29]		Zhou et al., 2015 [30]		Povlsen et al., 2014 [31]	
	LF	CCH	LF	CCH	LF	CCH	LF	CCH	LF	CCH
Patients (n)	26	52	14	38	19	18	66	66	10	10
Total complications (n)	2	9	0	11	10	26	10	140	10	2
Total no. of digits (n)	NR	NR	18	50	NR	NR	101	100	10	10
Total no. of joints (n)	109	198	32	86	77	29	91	86	10	10
Tenosynovitis	NR	NR	NR	NR	NR	NR	3	0	NR	NR
Wound dehiscence	NR	NR	NR	NR	NR	NR	2	0	NR	NR
Neurapraxia/Nerve injury	NR	NR	NR	NR	NR	NR	2	0	NR	NR
Edema/Swelling	NR	NR	NR	NR	0	6	NR	49	NR	NR
Bleeding	NR	NR	0	3	NR	NR	NR	NR	NR	NR
Axillary pain/Tenderness	NR	NR	NR	NR	NR	NR	NR	6	NR	NR
Pain in extremity	NR	NR	NR	NR	NR	NR	NR	17	NR	NR
Injection site blisters/Vesicles	0	1	NR	NR	NR	NR	NR	9	NR	NR
Pruritis/Rash	NR	NR	NR	NR	NR	NR	NR	3	NR	NR
Contusion/Bruising/Hematoma/Ecchymosis	NR	NR	NR	NR	1	6	NR	42	NR	NR
Erythema	NR	NR	NR	NR	NR	NR	0	3	NR	NR
Paraesthesia/Numbness	1	1	NR	NR	6	0	3	3	NR	NR
Pain/Scar tenderness	NR	NR	NR	NR	2	9	NR	NR	NR	NR
Scar sequelae (Hypertrophic scar, Skin laceration, tear, or fissure)	1	5	NR	NR	1	5	0	5	10	2
Per scandium wound healing	NR	NR	NR	5	NR	NR	NR	NR	NR	NR
Flare reaction	NR	NR	NR	NR	NR	NR	0	1	NR	NR
Lymphadenopathy/Lymphangitis	NR	NR	0	3	NR	NR	NR	2	NR	NR
Arthritis of the finger	0	1	NR	NR	NR	NR	NR	NR	NR	NR
Pneuomonia	0	1	NR	NR	NR	NR	NR	NR	NR	NR

To compare the complications of LF and CCH injections among these five studies, the most common complication seen in LF was scar sequelae (12 out of 32, 37.5%), while in CCH injection it was contusion/bruising/hematoma/ecchymosis (43 out of 188, 22.87%).

**Figure 2:**
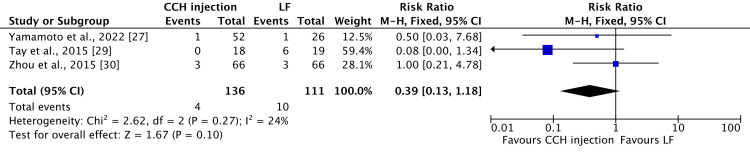
A forest plot with a 95% confidence interval compares the occurrence of paraesthesia/numbness in collagenase injections versus limited fasciectomy. Three studies [27,29,30] are included. CCH: collagenase *Clostridium histolyticum*; LF: limited fasciectomy; CI: confidence interval

Of note, two complications, specifically paraesthesia/numbness and scar sequelae (skin laceration, tear, fissure, or hypertrophic scar), were reported in three to four papers, respectively, facilitating the application of a meta-analysis. The fixed-effect model for paranesthesia/numbness in CCH injection vs. LF shows an RR of 0.39 (95% CI 0.13-1.18) with a p-value of 0.1 and low heterogeneity, as demonstrated in Figure 2.

**Figure 3:**
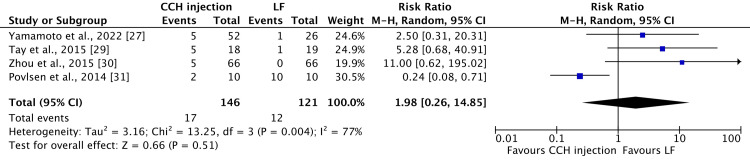
A forest plot with a 95% confidence interval compares the occurrence of scar sequelae (hypertrophic scar, skin laceration, tear, or fissure) in collagenase injections versus limited fasciectomy. High heterogeneity is noted (77%) and a random-effects model is used. Four studies [27,29-31] are included. CCH: collagenase *Clostridium histolyticum*; LF: limited fasciectomy; CI: confidence interval

However, for scar sequelae (skin laceration, tear, fissure, or hypertrophic scar) in CCH injection vs. LF, we noticed a high heterogeneity (77%), so we applied a random effect model. The result yielded an RR of 1.98 (95% CI 0.26-14.85) with a p-value of 0.51, as depicted in Figure 3.

**Figure 4:**
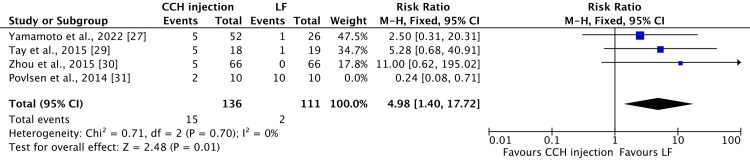
A forest plot was compared with a 95% confidence interval to compare the occurrence of scar sequelae (hypertrophic scar, skin laceration, tear, or fissure) in collagenase injections versus limited fasciectomy after excluding Povlsen et al. as a source of the heightened heterogeneity. Three studies are included [27,29,30], with the exclusion of Povlsen et al. [31]as a source of the heterogeneity. CCH: collagenase *Clostridium histolyticum*; LF: limited fasciectomy; CI: confidence interval

To explore the origin of this heightened heterogeneity, an attempt was made to exclude the study conducted by Povlsen et al. [31]. Interestingly, the heterogeneity decreased from 79% to 0%, revealing an RR of 4.98 (95% CI 1.40-17.72) and a p-value of 0.01, indicating statistical significance, as demonstrated in Figure 4.

Complications of Limited Fasciectomy

Regarding studies discussing LF [33, 34], a total of 250 patients were included, with one of the two studies reporting the involvement of 127 joints [34]. Both studies reported a total of 65 complications, with the most common complications being neuropraxia/nerve injury (30.76%), paraesthesia/numbness (20%), and scar sequelae such as hypertrophic scar, skin laceration, tear, or fissure (16.92%), as outlined in Table 3.

**Table 3:** Complications of limited fasciectomy NR: not reported

Articles	Zhou et al., 2016 [33]	van Rijssen et al., 2006 [34]
Patients (N)	194	56
Total complications (N)	48	17
Total no. of joints (N)	NR	127
Wound infection	3	1
Tenosynovitis	1	NR
Neurapraxia or nerve injury	19	1
Edema/Swelling	2	NR
Arterial injury	1	NR
Wound healing problems	6	NR
Stiffness	1	NR
Persistent pain	1	NR
Sympathetic dystrophia	1	0
Cold intolerance	1	NR
Contusion/Bruising/Hematoma/Ecchymosis	1	1
Paraesthesia/Numbness	NR	13
Scar sequelae (hypertrophic scar, skin laceration, tear, or fissure)	11	NR
Changed Semmes-Weinstein monofilament	NR	1

Complications of Injectable Collagenase

Meanwhile, the total number of patients involved in the six CCH injection studies was 352 [26,35-39]. Among these studies, only five reported a combined total of 872 complications across 360 joints in 307 patients [26,35-37,39]. The most common complications observed in these patients included contusion/bruising/hematoma/ecchymosis (21.9%), edema/swelling (16.74%), and inflammatory pain (14.44%), as detailed in Table 4.

**Table 4:** Complications of collagenase injections NR: not reported

Articles	Kuboi et al., 2021 [26]	Alberton et al., 2013 [35]	Abe et al., 2019 [36]	Sanjuan-Cerveró et al., 2018 [37]	Basso et al., 2023 [39]
Patients (N)	14	40	36	151	66
Total complications (N)	3	27	95	730	17
Total no. of joints (N)	14	40	46	194	66
Edema/Swelling	NR	2	36	108	NR
Injection site blisters/Vesicles or epidermal necrosis	NR	4	8	53	NR
Pruritis/Rash	NR	NR	NR	53	NR
Contusion/Bruising/Hematoma/Ecchymosis	1	9	31	150	NR
Pain/Scar tenderness	NR	NR	NR	86	NR
Scar sequelae (skin laceration, tear, fissure, or hypertrophic scar)	2	9	9	56	17
Inflammatory pain	NR	NR	NR	126	NR
Lymphadenopathy/Lymphangitis	NR	3	11	30	NR
Pain during extension	NR	NR	NR	68	NR

Patient Satisfaction

Patient satisfaction was evaluated in four of the six comparative studies [27,28,30,32], employing diverse metrics such as the mean first and final Quick DASH follow-up score, change in MHQ satisfaction score, pre-and post-operative MHQ satisfaction, and the mean first and final Hand 10 scores, as seen in Table 5.

**Table 5:** Patient satisfaction following injectable collagenase versus limited fasciectomy was seen in the comparative studies. Quick DASH: Quick Disabilities of the Arm, Shoulder, and Hand; MHQ: Michigan Hand Outcomes Questionnaire; CCH: collagenase *Clostridium histolyticum*; LF: limited fasciectomy; NR: not reported

Articles	Yamamoto et al., 2022 [27]			Leclère et al., 2018 [28]			Zhou et al., 2015 [30]			Naam et al., 2013 [32]		
	CCH	LF	P-value	CCH	LF	P-value	CCH	LF	P-value	CCH	LF	P-value
First follow-up Quick DASH score, Mean	NR	NR	NR	NR	NR	NR	NR	NR	NR	3 (1–34)	38 (30–60)	0.002
Final follow-up Quick DASH score, Mean	NR	NR	NR	NR	NR	NR	NR	NR	NR	3 (0–30)	3 (0–35)	0.6
The change in MHQ satisfaction scores	NR	NR	NR	NR	NR	NR	19.8	4.2	0.003	NR	NR	NR
Pre-operative MHQ satisfaction score, % (range)	NR	NR	NR	93 (75–100)	92 (75–100)	NR	NR	NR	NR	NR	NR	NR
Post-operative MHQ satisfaction score, % (range)	NR	NR	NR	96 (79–100)	97 (92–100)	NR	NR	NR	NR	NR	NR	NR
Pre-treatment Hand 10 score, Mean ± SD	15.5 ± 18.2	14.2 ± 15.4	0.76	NR	NR	NR	NR	NR	NR	NR	NR	NR
First follow-up Hand 10 score, Mean ± SD	25.2±23.7	46.1±23.8	<0.01	NR	NR	NR	NR	NR	NR	NR	NR	NR
Final follow-up Hand 10 score, Mean ± SD	6.5 ± 10.3	7.9 ± 11.3	0.59	NR	NR	NR	NR	NR	NR	NR	NR	NR

Due to the utilization of distinct scoring systems in each study, direct comparisons are precluded. However, it's worth noting that, as indicated by Yamamoto et al. [27], Zhou et al. [30], and Naam et al. [32], CCH injection has demonstrated superior patient satisfaction, particularly notable during the initial follow-up after the procedure. Interestingly, a comparable improvement in satisfaction rates for both LF and CCH injections is observed at the final follow-up across all studies.

One of the two LF studies reported patient satisfaction using baseline and final follow-up Quick DASH scores, indicating an approximate return of the score to the pre-operative level, as demonstrated in Table 6 [34].

**Table 6:** Patient satisfaction seen following injectable collagenase or limited fasciectomy in the non-comparative studies. Quick DASH: Quick Disabilities of the Arm, Shoulder, and Hand; URAM: Unité Rhumatologique des Affections de la Main; EQ-VAS: EuroQol-visual analogue scales; CCH: collagenase *Clostridium* histolyticum; LF: limited fasciectomy; NR: not reported

Articles	CCH	LF	CCH		
	Kuboi et al., 2021 [26]	van Rijssen et al., 2006 [34]	Abe et al., 2019 [36]	De Vitis et al., 2020 [38]	Basso et al., 2023 [39]
Baseline Quick DASH score, Mean ± SD	NR	14±12	16	23.6 ± 5.2	22 ± 17.3
Final follow-up Quick DASH score, Mean ± SD	NR	16	4.5	8.4 ± 4.1	10.45 ±13
Baseline URAM score	NR	NR	10.4	NR	NR
Final follow-up URAM score	NR	NR	2.9	NR	NR
Baseline Hand 10 score, Mean ± SD	15.5 ± 16.1	NR	NR	NR	NR
Final follow-up Hand 10 score, Mean ± SD	5.3 ± 8.0	NR	NR	NR	NR
Baseline EQ-VAS score	67.7 ± 24.6	NR	NR	NR	NR
Final follow-up EQ-VAS score	88.1 ± 14.5	NR	NR	NR	NR

In four out of the six CCH injection studies [26,36,38,39], patient satisfaction was measured using baseline and final follow-up Quick DASH, URAM, Hand 10, and EQ-VAS scores, consistently revealing increased patient satisfaction across all scores as outlined in Table 6. In particular, the mean baseline Quick DASH score for 141 patients in three of the CCH injection studies was 20.91, with a mean final follow-up score of 8.36, signifying a significant improvement in satisfaction (39.9%), regardless of the follow-up duration.

Risk of Bias and Quality Assessment

Two independent reviewers concurrently evaluated the risk of bias in the included studies. They employed the MINORS tool for non-randomized studies, as detailed in Tables 7-8, and the RoB 2 tool for randomized studies, as illustrated in Table 9.

**Table 7:** Review authors' judgments about each risk of bias item for each included comparative study according to the methodological index for non-randomized studies (MINORS) assessment tool.

Item	Yamamoto et al., 2022 [27]	Leclère et al., 2018 [28]	Tay et al., 2015 [29]	Zhou et al., 2015 [30]	Povlsen et al.,2014 [31]	Naam et al., 2013 [32]
A clearly stated aim	2	2	2	2	2	2
Inclusion of consecutive patients	0	2	2	0	2	2
Prospective collection of data	2	2	2	2	2	0
Endpoints appropriate to the aim of the study	2	2	2	2	2	2
Unbiased assessment of the study endpoint	0	0	0	0	0	0
Follow-up period appropriate to the aim of the study	2	2	2	2	2	2
Loss to follow-up less than 5%	2	2	2	2	2	2
Prospective calculation of the study size	2	2	2	2	2	2
An adequate control group	2	2	2	2	2	2
Contemporary groups	2	2	2	2	2	2
Baseline equivalence of groups	1	2	2	2	2	2
Adequate statistical analyses	2	2	2	2	2	2
Total score	19	22	22	20	22	20

**Table 8:** Review authors' judgments about each risk of bias item for each included non-comparative study according to the methodological index for non-randomized studies (MINORS) assessment tool.

Item	Kuboi et al., 2021 [26]	Zhou et al., 2016 [33]	Alberton et al., 2015 [35]	Sanjuan-Cerveróet al., 2018 [37]	De Vitis et al., 2020 [38]	Basso et al., 2023 [39]
A clearly stated aim	2	2	2	2	2	2
Inclusion of consecutive patient	2	0	2	1	0	2
Prospective collection of data	2	2	2	2	2	2
Endpoints appropriate to the aim of the study	2	2	2	2	2	2
Unbiased assessment of the study endpoint	0	0	2	0	0	0
Follow-up period appropriate to the aim of the study	2	2	2	2	2	2
Loss to follow-up less than 5%	2	2	2	1	0	2
Prospective calculation of the study size	2	2	2	2	2	2
Total Score	14	12	16	12	10	14

**Table 9:** Review authors' judgments about each risk of bias item for each included randomized controlled trial according to the revised Cochrane risk of bias (RoB 2) assessment tool.

Studies	Bias arising from the randomization process	Bias due to deviations from intended interventions	Bias due to missing outcome data	Bias in measurement of the outcome	Bias in selection of the reported result	Overall RoB
van Rijssen et al., 2006 [34]	Some concerns	Some concerns	Low	Low	Low	Some concerns
Abe et al., 2019 [36]	Low	Some concerns	Low	Low	Low	Some concerns

In the MINORS tool assessment, the total scores ranged from 10 to 22, with an average score of 15.75. The items with the lowest scores were the unbiased assessment of the study endpoint, where most studies received a score of 0, except for one, and the inclusion of a consecutive series of patients, which scored one or two in the majority of studies. Conversely, the items with the highest scores included the clearly stated aim of the study (score of two in all studies), prospective calculation of the study size (score of two in all studies), appropriate follow-up period (score of two in all studies), and clearly defined endpoints (score of two in all studies). With regards to the RoB 2 tool, the two included RCTs were found to have some concerns regarding their risk of bias, primarily in the domain of bias due to deviations from intended interventions.

Discussion

Dupuytren's disease is a fibroproliferative disorder affecting the hand fascia, marked by abnormal myofibroblast growth primarily consisting of type III collagen and influenced by cytokines [1]. It progresses through proliferative, involutional, and residual phases, involving fibroplasia, nodule formation, and uncontrolled fibroblast proliferation triggered by local mediators. In the involution phase, cell rearrangement induces collagen synthesis, contraction, and subsequent contractures [5]. The residual phase witnesses the nodules regressing into thick cords, resulting in visible cords and severe contractures [6]. If left untreated, these contractures may lead to deformities, causing a significant decline in the patient's overall quality of life [3,40,41]. Among the various treatment options available, LF and CCH injections have garnered significant attention [12]. In this systematic review and meta-analysis, we aim to compare complications and patient satisfaction related to these treatments, with the ultimate goal of offering clinicians valuable insights to select a safer and more effective approach to managing DD.

Our study comprises data from 967 patients, with a total of 1,344 reported treated joints, for an average follow-up duration of 19.22 (1-84.06) months. Among these, 498 joints were treated with LF and 846 with CCH injections. Remarkably, among the 491 patients who received CCH treatment, 1,060 complications were reported, with an average of approximately 2.15 complications per patient. Aligning with two existing literatures [10,42], the predominant complications observed in patients undergoing CCH injections were contusion/bruising/hematoma/ecchymosis (22.54% of 1,060 reported complications), edema/swelling (18.96% of 1,060 reported complications), and inflammatory pain (11.8% of 1,060 reported complications). Despite the relatively high incidence of complications (215% per patient) following CCH injection, it is noteworthy that all these complications are mild. As also observed by Cooper et al. [42], these complications are expected to be temporary, resolving over time without causing functional impairment. Conversely, among the 385 patients who underwent LF treatment, only 97 complications were documented, accounting for 0.25 complications per patient. The most common complications in this group were paraesthesia/numbness (23.7% of 97 reported complications), scar sequelae such as skin laceration, tear, fissure, or hypertrophic scar (23.7% of 97 reported complications), and neurapraxia/nerve injury (22.6% of 97 reported complications). Once again, these findings are consistent with the existing literature [33,42]. It is crucial to emphasize that, despite the lower incidence per patient (25.2%), LF treatment yielded fewer reported complications compared to CCH injections. However, it is noteworthy that the reported complications for LF treatment tended to be more severe. Complications such as neurapraxia or nerve injury may involve longer-lasting effects, potentially resulting in more persistent symptoms and a profound impact on nerve function, leading to more substantial functional impairment. Our meta-analysis result on comparative studies indicates that paraesthesia or numbness is more commonly seen following CCH injection than LF (four patients out of 136 patients vs. 10 patients out of 111, respectively), as compared with scar sequelae (hypertrophic scar, skin laceration, tear, or fissure), which exhibit an opposite pattern (17 patients out of 146 vs. 12 patients out of 121, respectively), without statistical significance. Notably, during the analysis, a substantial heterogeneity of 77% was observed in the context of scar sequelae (hypertrophic scar, skin laceration, tear, or fissure). Upon excluding the source of this heterogeneity, the outcome revealed a statistically significant association, indicating that scar sequelae (hypertrophic scar, skin laceration, tear, or fissure) are more commonly observed with CCH, with a p-value of 0.01.

Measuring patient satisfaction across the studies is quite challenging, as each study employed different satisfaction assessment tools. Moreover, some studies used different assessment methods. Due to this variation, we are not able to provide a clear assessment of patient satisfaction. Nevertheless, in line with existing literature [27,30,32,42], it can be inferred that patients undergoing CCH injection experience an earlier improvement in satisfaction, while both CCH injection and LF show similar long-term improvement in satisfaction using various assessment tools. This suggests that both treatment modalities are associated with favorable levels of patient satisfaction. According to Shaw et al., CCH injections are seen to be more effective in mild and moderate disease [43], unlike LF, which is seen to be more beneficial in aggressive and recurrent disease [44,45].

Therefore, we can conclude that CCH injection may be more suitable for patients with mild to moderate disease seeking early improvement post-procedure, while LF may be preferable for those tolerant of delayed improvement and with a more severe degree or recurrent form of DD. This study offers valuable insights into complications and patient satisfaction. However, it is important to address certain limitations, such as the limited number of comparative studies. Furthermore, our study features a larger sample of patients who underwent CCH injections compared to those who underwent LF, potentially introducing bias into our analysis. Moreover, our analysis did not separately examine several factors, such as patient diathesis, baseline disease severity, the affected joint type (e.g., MCP or PIP), and the involvement of multiple digits, which can influence the frequency of complications. Therefore, the complication rates in this study are derived from a diverse patient population, and direct comparisons with a specific subset may not be feasible. Furthermore, no sensitivity analysis was performed to determine the impact of additional confounding variables on treatment response, including age, body mass index (BMI), and chronic illnesses such as diabetes, and hypertension. Future research should aim for larger sample sizes, conduct comprehensive analyses of patient satisfaction, and broaden the scope of meta-analysis by refining inclusion criteria to incorporate non-English studies.

## Conclusions

In conclusion, the analysis of available studies reveals that both treatments have associated complications, with CCH injection showing a higher number of reported complications compared to LF. Notably, CCH complications are generally mild, contrasting with LF, which, while having fewer complications, tends to exhibit more severe ones such as neuropraxia or nerve injury. Specifically, our meta-analysis of the comparative studies showed that there are no statistically significant differences in the occurrence of paresthesia or numbness following CCH injection and LF. However, a statistically significant association was observed between skin laceration, tear, or fissure and CCH injection, with a p-value of 0.01. Both treatment options demonstrate an overall improvement in patient satisfaction at the final follow-up, but CCH injection is associated with an earlier satisfaction improvement.

In clinical practice, we recommend clinicians consider CCH injection for patients with mild to moderate disease seeking early improvement, considering the higher risk of mild complications. Limited fasciectomy may be preferable for those tolerant of delayed improvement or with a more severe or recurrent form of DD, considering the possibility of severe complications.

## References

[1] Khashan M, Smitham PJ, Khan WS, Goddard NJ (2011). Dupuytren’s disease: review of the current literature. Open Orthop J.

[2] Hindocha S (2018). Risk factors, disease associations, and Dupuytren diathesis. Hand Clin.

[3] Eckerdal D, Nivestam A, Dahlin LB (2014). Surgical treatment of Dupuytren's disease - outcome and health economy in relation to smoking and diabetes. BMC Musculoskelet Disord.

[4] Verjee LS, Midwood K, Davidson D, Essex D, Sandison A, Nanchahal J (2009). Myofibroblast distribution in Dupuytren's cords: correlation with digital contracture. J Hand Surg Am.

[5] Yvon A, Volk SW, Bayat A (2014). Superficial dermal and fascial fibromatoses. Pathobiology of Human Disease: A Dynamic Encyclopedia of Disease Mechanisms.

[6] Saraf S (2010). Dupuytren's disease. Indian J Dermatol Venereol Leprol.

[7] Bogdanov I, Rowland Payne C (2019). Dupuytren contracture as a sign of systemic disease. Clin Dermatol.

[8] Salari N, Heydari M, Hassanabadi M (2020). The worldwide prevalence of the Dupuytren disease: a comprehensive systematic review and meta-analysis. J Orthop Surg Res.

[9] Al-Qattan M (2000). Dupuytren’s disease in Saudi Arabia. Can J Plast Surg.

[10] Hovius SE, Zhou C (2018). Advances in minimally invasive treatment of Dupuytren disease. Hand Clin.

[11] Ball C, Izadi D, Verjee LS, Chan J, Nanchahal J (2016). Systematic review of non-surgical treatments for early dupuytren's disease. BMC Musculoskelet Disord.

[12] Denkler KA, Park KM, Alser O (2022). Treatment options for Dupuytren’s disease: tips and tricks. Plast Reconstr Surg Glob Open.

[13] Dias JJ, Aziz S (2018). Fasciectomy for Dupuytren contracture. Hand Clin.

[14] Denkler K (2010). Surgical complications associated with fasciectomy for dupuytren's disease: a 20-year review of the English literature. Eplasty.

[15] (2014). Collagenase Clostridium histolyticum for Dupuytren’s contracture. Australian Prescriber.

[16] Yoshida R, Baron S, Rodner C, Ferreira J (2019). Biologics in hand surgery. Biologics in Orthopaedic Surgery.

[17] Chen NC, Srinivasan RC, Shauver MJ, Chung KC (2011). A systematic review of outcomes of fasciotomy, aponeurotomy, and collagenase treatments for Dupuytren's contracture. Hand (N Y).

[18] Moher D, Liberati A, Tetzlaff J, Altman DG (2009). Preferred reporting items for systematic reviews and meta-analyses: the PRISMA statement. PLoS Med.

[19] Ouzzani M, Hammady H, Fedorowicz Z, Elmagarmid A (2016). Rayyan-a web and mobile app for systematic reviews. Syst Rev.

[20] Slim K, Nini E, Forestier D, Kwiatkowski F, Panis Y, Chipponi J (2003). Methodological index for non-randomized studies (minors): development and validation of a new instrument. ANZ J Surg.

[21] Sterne JA, Savović J, Page MJ (2019). RoB 2: a revised tool for assessing risk of bias in randomised trials. BMJ.

[22] Gummesson C, Ward MM, Atroshi I (2006). The shortened disabilities of the arm, shoulder and hand questionnaire (QuickDASH): validity and reliability based on responses within the full-length DASH. BMC Musculoskelet Disord.

[23] Nolte MT, Shauver MJ, Chung KC (2017). Normative values of the Michigan hand outcomes questionnaire for patients with and without hand conditions. Plast Reconstr Surg.

[24] Beaudreuil J, Allard A, Zerkak D (2011). Unité rhumatologique des affections de la main (Uram) scale: development and validation of a tool to assess Dupuytren’s disease-specific disability. Arthritis Care Res (Hoboken).

[25] Feng Y, Parkin D, Devlin NJ (2014). Assessing the performance of the EQ-VAS in the NHS PROMs programme. Qual Life Res.

[26] Kuboi T, Tajika T, Endo F (2021). Collagenase Clostridium histolyticum injection therapy improves health-related quality of life in patients with Dupuytren’s disease. Prog Rehabil Med.

[27] Yamamoto M, Yasunaga H, Kakinoki R (2022). The CeCORD-J study on collagenase injection versus aponeurectomy for Dupuytren's contracture compared by hand function and cost effectiveness. Sci Rep.

[28] Leclère FM, Kohl S, Varonier C, Unglaub F, Vögelin E (2018). Range of motion, postoperative rehabilitation and patient satisfaction in MCP and PIP joints affected by Dupuytren Tubiana stage 1-3: collagenase enzymatic fasciotomy or limited fasciectomy? A clinical study in 52 patients. Arch Orthop Trauma Surg.

[29] Tay TK, Tien H, Lim EY (2015). Comparison between collagenase injection and partial fasciectomy in the treatment of Dupuytren’s contracture. Hand Surg.

[30] Zhou C, Hovius SE, Slijper HP, Feitz R, Van Nieuwenhoven CA, Pieters AJ, Selles RW (2015). Collagenase Clostridium histolyticum versus limited fasciectomy for Dupuytren’s contracture: outcomes from a multicenter propensity score matched study. Plast Reconstr Surg.

[31] Povlsen B, Shields AM, Bhabra GS (2014). Resource utilisation associated with single digit Dupuytren's contracture treated with either surgery or injection of collagenase Clostridium histolyticum. Hand Surg.

[32] Naam NH (2013). Functional outcome of collagenase injections compared with fasciectomy in treatment of Dupuytren's contracture. Hand (N Y).

[33] Zhou C, Hovius SE, Slijper HP, Zuidam MJ, Smit X, Feitz R, Selles RW (2016). Predictors of patient satisfaction with hand function after fasciectomy for Dupuytren’s contracture. Plast Reconstr Surg.

[34] van Rijssen AL, Gerbrandy FS, Ter Linden H, Klip H, Werker PM (2006). A comparison of the direct outcomes of percutaneous needle fasciotomy and limited fasciectomy for Dupuytren's disease: a 6-week follow-up study. J Hand Surg Am.

[35] Alberton F, Corain M, Garofano A, Pangallo L, Valore A, Zanella V, Adani R (2014). Efficacy and safety of collagenase Clostridium histolyticum injection for Dupuytren contracture: report of 40 cases. Musculoskelet Surg.

[36] Abe Y (2020). Comparison of treatment outcomes after collagenase injection and percutaneous needle fasciotomy for Dupuytren’s contracture: objective and subjective comparisons with a 3-year follow-up. Plast Reconstr Surg.

[37] Sanjuan-Cerveró R, Carrera-Hueso FJ, Vazquez-Ferreiro P (2018). Adverse effects associated with collagenase clostridium histolyticum in Dupuytren disease: a prospective study. Orthop Traumatol Surg Res.

[38] De Vitis R, Passiatore M, Perna A, Careri S, Cilli V, Taccardo G (2020). Seven-year clinical outcomes after collagenase injection in patients with Dupuytren's disease: a prospective study. J Orthop.

[39] Basso MA, Bernasconi A, Balato G, Cozzolino A, Famiglietti G, Smeraglia F (2023). Clinical results of collagenase treatment for Dupuytren’s disease: a case series study with 2-years follow-up. Acta Ortop Bras.

[40] Aykut S, Baydar M, Büyük AF, Öztürk İA, Özden E, Öztürk K (2017). Surgical treatment results for dupuytren's disease. Acta Ortop Bras.

[41] Karam M, Kahlar N, Abul A, Rahman S, Pinder R (2022). Comparison of hand therapy with or without splinting post fasciectomy for Dupuytren’s contracture: systematic review and meta-analysis. J Hand Microsurg.

[42] Cooper TB, Poonit K, Yao C, Jin Z, Zheng J, Yan H (2020). The efficacies and limitations of fasciectomy and collagenase clostridium histolyticum in Dupuytren's contracture management: a meta-analysis. J Orthop Surg (Hong Kong).

[43] Shaw RB Jr, Chong AK, Zhang A, Hentz VR, Chang J (2007). Dupuytren's disease: history, diagnosis, and treatment. Plast Reconstr Surg.

[44] Ashdown T, Hayter E, Morris JA, Clough OT, Little M, Hardman J, Anakwe RE (2021). Repeat limited fasciectomy is a safe and effective treatment for recurrence of Dupuytren's disease. Bone Joint J.

[45] Könneker S, Broelsch GF, Krezdorn N, Dastagir K, Kuhbier JW, Paprottka FJ, Vogt PM (2017). Multiple recurrences in aggressive forms of Dupuytren’s disease-can patients benefit from repeated selective fasciectomy?. Plast Reconstr Surg Glob Open.

